# Accelerated PAH Transformation in the Presence of Dye Industry Landfill Leachate Combined with Fungal Membrane Lipid Changes

**DOI:** 10.3390/ijerph192113997

**Published:** 2022-10-27

**Authors:** Aleksandra Góralczyk-Bińkowska, Andrzej Długoński, Przemysław Bernat, Jerzy Długoński, Anna Jasińska

**Affiliations:** 1 Department of Industrial Microbiology and Biotechnology, Faculty of Biology and Environmental Protection, University of Lodz, Banacha 12/16 Street, 90-237 Lodz, Poland; 2 Institute of Biological Sciences, Faculty of Biology and Environmental Sciences, Cardinal Stefan Wyszyński University in Warsaw, Wóycickiego 1/3 Street, 01-938 Warsaw, Poland; 3 Institute of Ecology and Environmental Protection, Faculty of Biology and Environmental Protection, University of Lodz, Banacha 12/16 Street, 90-237 Lodz, Poland

**Keywords:** *Nectriella pironii*, PAHs, leachates, biodegradation, lipidomics

## Abstract

The ascomycete fungus *Nectriella pironii*, previously isolated from soil continuously contaminated by dye industry waste, was used for the biodegradation of phenanthrene (PHE), benz[a]anthracene (B[a]A), and benz[a]pyrene (B[a]P). The degradation of polycyclic aromatic hydrocarbons (PAHs) by *N. pironii* was accelerated in the presence of landfill leachate (LL) collected from the area of fungus isolation. The rate of cometabolic elimination of PHE and B[a]P in the presence of LL was, respectively, 75% and 94% higher than in its absence. LC-MS/MS analysis revealed that PAHs were converted to less-toxic derivatives. The parallel lipidomic study showed changes in membrane lipids, including a significant increase in the content of phosphatidylcholine (PC) (almost double) and saturated phospholipid fatty acids (PLFAs) and a simultaneous reduction (twofold) in the content of phosphatidylethanolamine (PE) and unsaturated PLFAs, which may have promoted the fungus to PHE + LL adaptation. In the presence of PHE, an intense lipid peroxidation (fivefold) was observed, confirming the stabilization of the cell membrane and its extended integrity. Determining the course of elimination and adaptation to harmful pollutants is essential for the design of efficient bioremediation systems in the future.

## 1. Introduction

Rapid industrial and commercial growth in countries around the world has led to an alarming increase in the generation of municipal and industrial solid waste. In the European Union, the total waste generated by economic and household activities in 2018 was 2337 million tonnes, of which 101.7 million (4.4% of the total) was hazardous waste (HW). Since 2010, the total amount of HW generated has increased by 11.9%. The main sources of HW are agricultural fields, industries, domestic facilities, mining, and mineral-processing sites, as well as the natural environment. Only half of the generated HW is recovered and reused as fuel for producing electricity, while the remaining is landfilled [1].

Inappropriate management and disposal of HW landfills can pose a serious threat to human health and the environment. Landfill waste undergoes several physicochemical and biological processes that result in leachate. The composition of leachate depends mainly on the types of waste and residual moisture content in the landfill, the water filtration rate, the degradation stage, and the landfilling technology. Most leachates have a high concentration of dissolved organic matter, inorganic macrocomponents, and heavy metals and organic xenobiotics [2]. When leachate reaches the soil, surface, and groundwater around the landfill, the pollutants present in it can adversely affect ecosystems and public health. Leachate is often transported to the wastewater treatment plant (WWTP) for management; however, it is generally not treated adequately and poses a risk to the surface waters that receive the effluent.

Polycyclic aromatic hydrocarbons (PAHs) are a major group of contaminants in the leachate derived from HW landfills. These are present in coal and crude oil and are released during the combustion of fossil fuels. They are also found as a component in different chemicals, including textile dyes, resins, plastics, pesticides, and pharmaceuticals, or as intermediates during their transformation [3]. As a result, PAHs can be detected in various ecosystems, such as soil [4,5,6], surface and groundwater [7,8], and air [9]. It was reported that the content of 16 PAHs in the soils around the chemical plant n Shanxi (China) amounted to 42.5 mg kg^−1^ [10]. Torres-Farradá et al. [11] noted that the half-life of PAHs in soils is 5.7 to 9.1 years. As a result of their hydrophobic nature, PAHs easily bind to solids in wastewater and accumulate in the sludge. Their presence was observed in sludge released from textile dye plants [12,13] and leachate from landfills [14,15,16,17]. The concentration of PAHs in leachate samples could vary (between ng L^−1^ and μg L^−1^) as these samples have undesirable dissolved organic matter [18].

The toxicity, mutagenicity, and carcinogenicity of PAHs have been confirmed by several authors and also previously reviewed by Idowu et al. [19] and Patel et al. [20]. Based on available data on the possible human exposure to PAHs, as well as their toxicity and frequency of occurrence at HW sites, the United States Environmental Protection Agency (USEPA) has classified 16 PAHs as priority pollutants. These compounds include benz[a]anthracene, benz[a]pyrene, and phenanthrene [21]. Because PAHs are relatively poorly soluble in water and highly lipophilic, they can easily bioaccumulate in lipid tissue and remain persistent and resistant to biodegradation [22]. The selection of appropriate methods for PAH remediation is crucial and strongly dependent on the kind of the polluted matrix and the state of the environment. It is noteworthy that particular PAHs are characterized by diverse physical and chemical properties, which complicate the development of remediation techniques for PAH-contaminated sites. Hence, the exploitation of real matrices (soil or sewage) in studies regarding the removal of PAHs contributes to better predictions of their action.

Currently, available approaches for PAH treatment are classified into three groups: physical, chemical, and biological. Due to the hydrophobicity of PAHs, the most suitable method for their removal (especially in the case of high-molecular-weight PAHs (HMW-PAHs)) seems to be soil washing with a solvent (e.g., acetone, alcohol, hexane, dichloromethane, methyl ethyl ketone, toluene) [23]. The addition of surfactants may enhance the efficiency of soil washing by changing the solubility of PAHs, which, on the other hand, is highly dependent on some factors such as soil composition, the characteristics of PAHs, and the structure of the surfactant [24]. Among the physical methods for PAH removal from water, different membrane filtration approaches such as ultrafiltration, micro-/nanofiltration, and reverse osmosis or the application of several adsorbents, e.g., activated carbon, charcoal, biochar, magnetic nanomaterials, and graphene oxide have been used [25,26]. The most frequently described chemical methods are oxidation processes through common oxidants such as the Fenton reagent, potassium permanganate, sodium persulfate, and hydrogen peroxide [27].

One of the biological approaches to PAH oxidation is bioremediation through microbial enzymes such as oxygenases, dehydrogenases, lignin peroxidases, manganese peroxidases, laccases, and phenol oxidases. The reactions catalyzed by these enzymes are characterized by extreme efficiency in a wide range of temperatures and pH. On the other hand, for the isolation and purification of these enzymes from bacteria, fungi, and other living organisms, specific conditions and apparatus are necessary, which generates costs [20]. Moreover, free cells (FC) and immobilized cells (IC) find application in PAH biodegradation. For example, Partovinia and Naeimpoor [28] showed the entire biodegradation of PHE (250 ppm) after 7 days using FC and IC of a microbial consortium (screened from the Tehran Oil Refinery activated sludge) in polyvinyl alcohol cryogel beads. A promising biological method of PAH elimination from contaminated environments is microbial degradation, which is influenced by the number and type of the microorganisms, conditions of the environment, and chemical structure of degraded chemicals. In the literature, there are more studies regarding PAH elimination by bacteria than by fungi. Ligninolytic as well as nonligninolytic fungi show the ability to oxidize PAHs through the secretion of extracellular enzymes [29,30]. In many reviews, it can be found that the advantage of biological methods over physicochemical methods is, above all, their low cost and the fact that by-products characterized by higher toxicity than the parent compounds are produced during biodegradation [31]. Nevertheless, biological methods are ineffective at high concentrations of PAHs; hence, in the first stage of pollutant elimination, physical/chemical methods are used to reduce the concentration of chemicals to an optimal level for further biological processes and to increase the rate of biodegradation.

Several microbial species are known to degrade PAHs, and most of them—isolated and recovered from contaminated soil or sediments—show great potential against the contaminants. Although many investigations have focused on bacteria species, for example, *Mycobacterium* [32], *Nocardia* [33], and *Sphingomonas* [34] involved in the degradation of PAHs as sole sources of carbon and energy, it has also been found that fungi have the ability to mineralize toxic xenobiotics, especially to degrade HMW-PAHs in contaminated soils [35,36]. Besides the biosynthesis of enzymes, an advantage of using fungi is their tolerance to extreme and fluctuating environments [37,38]. According to Medura et al. [39], Ascomycete fungi belonging to the class Sordariomycetes, as well as the phylum Zygomycota, which are widely distributed in the environment and in polluted soils, have adapted to metabolize a broad spectrum of organic compounds, including PAHs.

This work demonstrates the ability of the ascomycete fungus *Nectriella pironii* IM 6443 to convert PHE, B[a]A, and B[a]P. The fungus was isolated from soil contaminated with deleterious industrial dye, which has previously been shown to retain its activity in the presence of landfill leachate (LL) and to eliminate azo dyes and aromatic amines [40,41]. Both the isolation of the fungus from contaminated areas and its application in biodegradation are based on the adaptation of the microorganism to unfavorable conditions. The present work showed that *N. pironii* IM 6443 can efficiently degrade particular PAHs in the presence of leachate obtained from a landfill where HW from dye industry plants was disposed of. The formed metabolites were identified by gas chromatography–tandem mass spectrometry analysis. A lipidomic study was conducted to understand the mechanisms that allow the fungus to survive in difficult environmental conditions and to remove PAHs from the growth environment From the application point of view, an extremely important part of this work was the use of landfill leachates in the research on the removal of PAHs as real environmental matrices. Another significant aspect was the choice of microorganism, which, due to the growth in soil continuously contaminated by deleterious waste, has developed mechanisms of adaptation and survival. The results of this work are expected to make a significant contribution to solving the problem of hazardous waste landfill management.

## 2. Materials and Methods

### 2.1. Reagents and LL

LL samples (L1 and L2) collected (according to ISO 5667-10 standard) from the landfill of the former “Boruta” Dye Industry Plant in Zgierz (Zgierz, Poland), then transported and stored with accordance to the ISO 5667-3:2018 standard, were kindly provided by the Voivodeship Inspectorate of Environmental Protection in Łódź, Poland. PHE, B[a]A, B[a]P, butylated hydroxytoluene (BHT), and thiobarbituric acid were purchased from Merck (Darmstadt, Germany). A stock solution (20 mg mL^−1^) of PHE was prepared in ethanol (POCH, Gliwice, Poland), and that of B[a]A and B[a]P in dimethyl sulfoxide (Merck, Germany). The phospholipid standards were obtained from Avanti Polar Lipids (Alabaster, AL, USA). The methanol, chloroform, and ethyl acetate solvents were purchased from Avantor (Gliwice, Poland). All chemicals were of high-purity grade.

### 2.2. Microorganism and Growth Conditions

The fungal strain *N. pironii* IM 6443 was isolated from a soil sample collected on the territory of the former “Boruta” Dye Industry Plant in Zgierz [40,41], and since that time, 20 passages were carried out. The strain was maintained on ZT slants (4 g of glucose, 4 g of Difco yeast extract, 25 g of agar, 0.5 L of wort (12° Blg), up to 1 L of H_2_O; pH 6.6–7.0) at 4 °C in the strain collection of the Department of Industrial Microbiology and Biotechnology, University of Lodz (Poland) [42]. The spores obtained from 10-day-old cultures of *N. pironii* on ZT slants were inoculated into 25 mL of WHI medium [42]. Initial preculture was maintained under conditions previously described [41].

### 2.3. Growth of N. pironii in Medium with PAHs and LL

LL was centrifuged at 6000× *g* for 10 min at 4 °C and filtered (0.22 μm). Then, 2 mL of the preculture was inoculated into Sabouraud dextrose broth liquid medium (BioMaxima, Lublin, Poland) supplemented with 20% (*v*/*v*) LL and 20 mg L^−1^ of PAHs (PHE, B[a]A, B[a]P). Biotic controls (containing only the medium and biomass) and abiotic controls (containing the medium and PAHs or LL) were also prepared. Samples containing the same volume of ethanol or DMSO were also prepared (to exclude the inhibitory effect of solvents on fungal growth). All the prepared cultures were incubated in a rotary shaker (120 rpm) at 28 °C. The biomass was separated from the cultures by filtration under a slight vacuum and then dried at 105 °C. The dry weight of the fungus was estimated as described by Góralczyk-Bińkowska et al. [41].

### 2.4. Analytical Methods

#### 2.4.1. Extraction and Quantification of PAHs

After incubation, fungal culture (20 mL) was homogenized in Falcon tubes with 10 mL of ethyl acetate and glass beads using a ball mill (MM 400, Retsch, Haan, Germany) for 3 min. The samples were vigorously shaken and centrifuged at 6500× *g* for 10 min. After centrifugation, the upper layer was collected and transferred to a new Falcon tube. Next, 10 mL of ethyl acetate was added to the bottom phase and the extraction procedure was repeated. The samples were shaken and centrifuged again as described above. Then, the upper phase was collected and combined with the already-collected phase. The resulting mixtures were dehydrated with anhydrous ammonium sulfate and evaporated to dryness under reduced pressure. Before chromatographic analysis, the solvent-free residues were dissolved in 1 mL of ultrapure ethyl acetate.

The PAHs in the samples were identified and quantified by gas chromatography– mass spectrometry (GC–MS). Analysis was carried out in an Agilent Model 7890 gas chromatograph, equipped with a 5975C mass detector. The compounds were separated using an HP 5 MS methyl polysiloxane capillary column (30 m × 0.25 mm i.d. × 0.25 mm ft). The column temperature was maintained at 70 °C for 3 min, then increased to 250 °C at a rate of 10 °C min^−1^ and finally to 280 °C at a rate of 20 °C min^−1^, and maintained at 280 °C for 7 min. Helium was used as a carrier gas at a flow rate of 1 mL min^−1^. Split injection was performed at a port temperature of 250 °C. Identification of PAHs in the samples was carried out based on the retention time and abundance of quantification/confirmation ions in the authentic PAH standards (Merck, Germany) or based on the literature data.

#### 2.4.2. Phospholipid Extraction and Analysis

The phospholipids of *N. pironii* were extracted following the Folch method with some modifications suggested by Bernat et al. [43]. Briefly, 100 mg of fungal biomass was transferred to a 2 mL Eppendorf tube containing 0.66 mL of chloroform, 0.33 mL of methanol, and glass beads. The samples were homogenized using a ball mill (MM 400, Retsch, Germany) at 30 rpm for 3 min (each cycle for 1 min). The resulting mixture was transferred to another Eppendorf tube, in which 0.2 mL of deionized water was added. The sample was subsequently vortexed and centrifuged at 2000× *g* for 5 min. After separation into two layers, the bottom phase was collected, transferred to a new Eppendorf tube, evaporated, and stored at −20 °C. Before analysis, the extracts were dissolved in 1 mL of methanol.

Phospholipid analysis was performed by liquid chromatography–tandem mass spectrometry (LC–MS/MS), as described by Bernat et al. [43]. Measurement was carried out in an Agilent 1200 HPLC system (Agilent Technologies, Santa Clara, CA, USA) and a 4500 QTrap mass spectrometer (Sciex, Framingham, MA, USA). For this, 10 μL of the diluted sample was injected into a Kinetex C18 column (50 mm × 2.1 mm, particle size: 5 μm; Phenomenex, Torrance, CA, USA). Water (A) and methanol (B), both containing 5 mM ammonium formate, were used as mobile phases. The solvent gradient began at 70% B, and then after 0.25 min, reached 95% B in 1 min, and continued at 95% B for 7 min before returning to the initial solvent composition (70% B) in 2 min. The column temperature was maintained at 40 °C and the flow rate at 500 µL min^−1^. The settings applied to the mass spectrometer ion source were as follows: spray voltage –4500 V, curtain gas 25 psi, nebulizer gas 60 psi, auxiliary gas 50 psi, and temperature 600 °C. Data analysis was performed in Analyst™ version 1.6.2 software (https://sciex.com/products/software/analyst-software (accessed on 16 May 2022); Sciex, Framingham, MA, USA).

#### 2.4.3. Lipid Peroxidation Assay

The degree of lipid peroxidation was determined as the content of thiobarbituric acid reactive substances (TBARS) using a spectrophotometric method described by Yagi with a few modifications suggested by Słaba et al. [44]. Briefly, 0.5 g of fresh wet biomass was homogenized with 9 mL of deionized water and 50 μL of 7.2% BHT (in ethanol) in a ball mill (MM 400, Retsch, Germany) at 30 m s^−1^ for 3 min. Then, 1 mL of the mixture was transferred to a Falcon tube containing 2 mL of 15 mM thiobarbituric acid in 15% trichloroacetic acid. The mixture was vortexed and heated at 95 °C in a water bath for 30 min. After cooling, the samples were centrifuged at 2000× *g* for 15 min and incubated at room temperature for 10 min. The absorbance of the supernatant was measured using a SPECORD 200-222U313 spectrophotometer (Analytik Jena, Jena, Germany) at a wavelength (λ) of 531 nm, and the value of nonspecific absorption at λ = 600 nm was subtracted. The results of the lipid peroxidation analysis were presented as micromoles of TBARS calculated per gram of wet biomass.

### 2.5. Statistical Analysis

Data were analyzed in STATISTICA version 13.3 software (https://statistica.software.informer.com/13.3/ (accessed on 25 May 2022); StatSoft, Tulsa, OK, USA). All samples were prepared in triplicate and the experiments were performed twice. The results are presented as the mean ± standard deviation. The normality of the data distribution was verified using the Shapiro–Wilk test. Normal distributed data were analyzed using one-way analysis of variance with Tukey’s post hoc test. For results with a nonnormal distribution, the nonparametric Mann–Whitney U test was performed and the means were compared based on the p values for multiple comparisons.

## 3. Results and Discussion

### 3.1. Characteristics of the Landfill of the Former “Boruta” Dye Industry Plant and the LL

**Figure 1:**
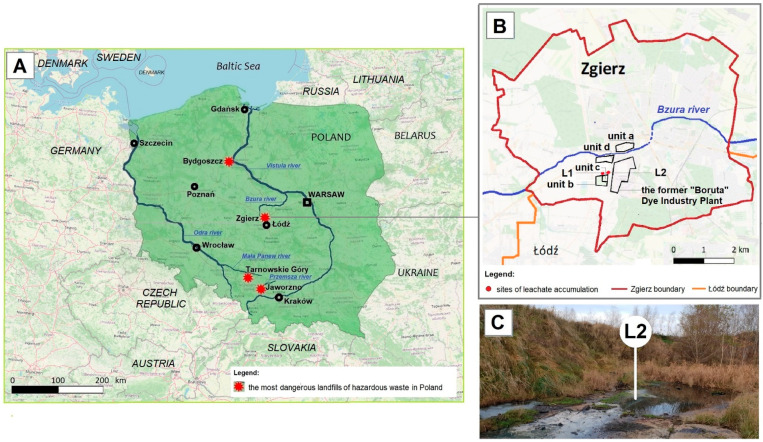
The most dangerous sources of industrial pollution in Poland (**A**) and location of the study region—the area of the former “Boruta” Dye Industry Plant and the landfill (**B**,**C**).

The HW landfill of the former “Boruta” Dye Industry Plant in Zgierz (Figure 1A) is one of the four most dangerous sources (known as hotspots) of industrial pollution in Poland that affects the blue-green infrastructure and the Baltic Sea [45,46,47]. In addition to having a high amount of different toxic chemicals, including PAHs and their metabolites, these four hotspots are located near water courses. By polluting groundwater, toxic chemicals from these hotspots enter nearby local rivers and subsequently the main Polish rivers, the Vistula and Oder, accumulating in the Baltic Sea, which is a cramped inland (415,266 km^2^) sea [48,49,50,51,52].

The total surface area of the landfill of the former “Boruta” Dye Industry Plant in Zgierz (Figure 1B) is 12 ha, which includes three units (marked as a, b, and c in Figure 1B). “Za Bzurą” which is the oldest landfill (Figure 1B, unit a), operating from the end of the 19th century up to 1995, has an area of 4.5 ha. This is followed by the ash, slag, and gypsum landfill (Figure 1B, unit b), with a surface area of 6.7 ha, which operated for about 10 years until 1996, and the last one “Kwatera I” (Figure 1B, unit c), with an area of 0.8 ha, in which a high amount of toxic industrial waste was deposited in the years 1995–2015 [41,53,54]. Although it occupies the smallest area, the last unit of the landfill poses the greatest threat to the environment. Due to improper slope protection, toxic leachates are not fully discharged into the municipal industrial WWTP (Figure 1B, unit d) and part of them accumulate in nearby areas (Figure 1C). Through groundwater, they pollute the Bzura and Vistula rivers one after another, and finally the Baltic Sea [54].

The main parameters of the leachates L1 and L2 that accumulate at the base of the landfill slope in the last years (2017–2021) are presented in Table 1. Throughout the period analyzed, the values of chemical oxygen demand (COD) and total organic carbon (TOC) were slightly and greatly higher, respectively, than the limits specified in the applicable standards, which is most likely due to the addition of municipal waste to the landfill [38].

Initially, petroleum hydrocarbon concentrations were at an acceptable level (below 15 mg L^−1^) in both leachates. However, since autumn 2020, they have increased significantly in leachate L1, exceeding the allowed values. In addition to petroleum hydrocarbons, leachates also had an increased amount of iron, which is most likely related to corrosion of the metal containers used to store industrial waste [53,55].

### 3.2. Growth of N. pironii in the Presence of PAHs and Elimination of PAHs

**Figure 2:**
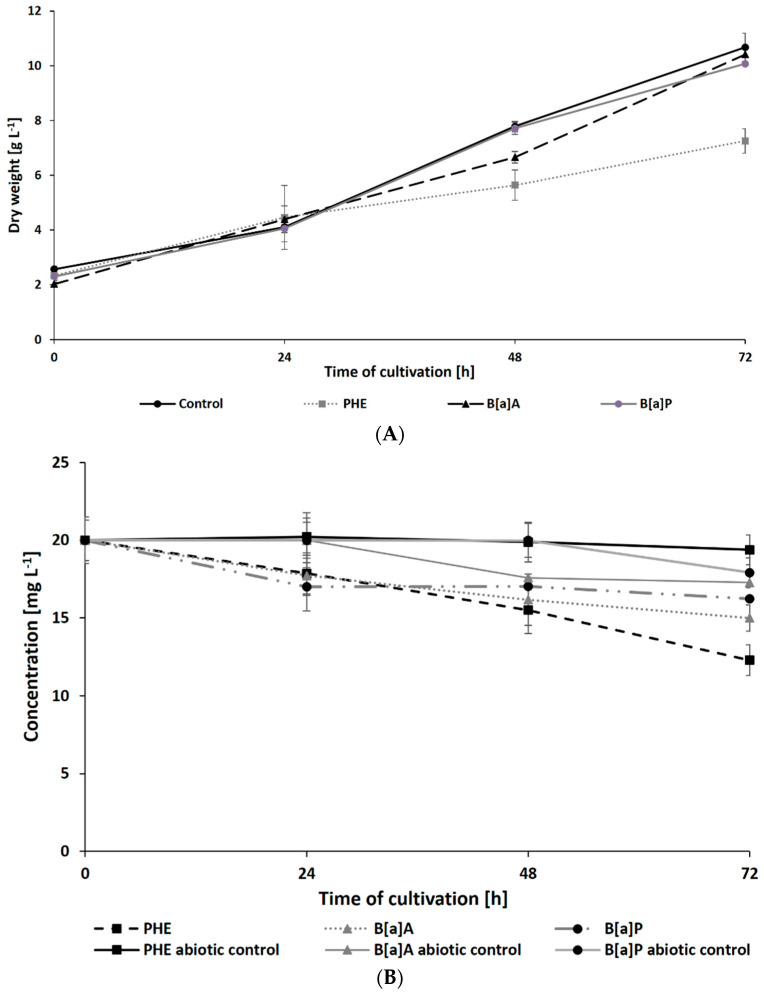
Dry weight of *N. pironii* (**A**) and PAH degradation of PAHs (**B**) during culture in Sabouraud medium containing PHE; B[a]A or B[a]P (20 mg L^−1^).

The results of *N. pironii* growth in Sabouraud medium supplemented with 20 mg L^−1^ of PHE, B[a]A, or B[a]P are presented in Figure 2A. In the presence of PHE, biomass production in the growth environment was reduced after 48 h. Due to the chemical structure of the PAHs used in this study and the fact that the resistance to biodegradation increases with the number of aromatic rings [56], it was expected that five-ring B[a]P would have the most toxic and inhibitory effect on fungal growth compared to three-ring PHE. However, the biomass content in the cultures containing B[a]A and B[a]P (10.07 ± 0.01 and 10.4 ± 0.24 g L^−1^, respectively) was similar to that of the control cultures. Parallelly, in cultures containing DMSO or ethanol (instead of PAH) to exclude their inhibitory effect, there were no significant differences in fungal growth.

Inhibition of *Penicillium* sp., *Talaromyces* sp., and *Hypoxylon* sp. growth in PHE-supplemented medium was previously reported by de Lima Souza et al. [57]. Lisowska et al. [58] also described that the spore germination of the filamentous fungus *Cunninghamella elegans* IM 1785/21Gp was limited by 70% after the addition of 25 mg L^−1^ of PHE, and the growth of the fungal was completely inhibited in the presence of a higher concentration of PHE (50–100 mg L^−1^). The data indicate that the tolerance of fungi to PAHs is influenced by various physical and chemical factors, including vapor pressure, solubility, and adsorption of these compounds [20]. Argumedo-Delira et al. [59] indicated a correlation between the growth of *Trichoderma* strains and factors such as vapor pressure and solubility of naphthalene, PHE, and B[a]P. According to Patel et al. [20] the vapor pressure of PHE, B[a]A, and B[a]P is equal to 6.8 × 10^−4^, 2.5 × 10^−6^, and 5.6 × 10^−9^ mmHg, respectively. Furthermore, the water solubility of PHE (1.1 mg L^−1^) is significantly higher than that of B[a]A (0.011 mg L^−1^) and B[a]P (0.0038 mg L^−1^). These characteristics of PHE can promote its adsorption in growth medium and facilitate its better attachment to the mycelium during cultivation [20,59]. It should also be noted that the fungus *N. pironii* used in this study was isolated from soil contaminated with pollutants released from the textile industry, including dyes, heavy metals, aromatic amines, and PAHs. This microorganism may have adaptive mechanisms that allow it to survive in unfavorable environmental conditions and exhibit metabolic activity observed in studies on a laboratory scale. The application of environmental isolates has also been widely described in the literature. For example, the ascomycetous fungus *Myrothecium roridum* IM 6482 has been shown to have a high potential to eliminate bisphenol A (BPA) [60] and textile dyes [61]. Janicki et al. [62] described that the filamentous fungus *Umbelopsis isabellina* IM 833 adapted to LL and degraded nonylphenol, 4-*tert*-octylphenol, 4-cumylphenol, and volatile phenols to less-toxic intermediates.

The present study also analyzed the ability of *N. pironii* IM 6443 to attack PHE, B[a]A, and B[a]P. During the first 24 h of incubation, a decrease in the concentration of the tested PAHs (initial concentration: 20 mg L^−1^) was observed (Figure 2B). After 72 h of incubation, the concentration of PHE, B[a]A, and B[a]P corresponded to 12.3 ± 0.99, 14.9 ± 0.84, and 16.2 ± 0.76 mg L^−1^, respectively. As shown in Table 2, PAH metabolites formed during the reaction were identified by GC-MS analysis. The mass spectra of the obtained intermediates are shown in Figure S1, Supplementary Materials. According to previous studies, the main mechanism through which fungi degrade PAHs is the intracellular accumulation of chemicals followed by their transformation [29]. In the present study, the presence of a protocatechuic acid-TMS derivative (trimethylsilyl), which confirms the generation of protocatechuic acid, was observed in cultures of *N. pironii* containing PHE, B[a]A, and B[a]P. Protocatechuic acid plays an important role in various metabolic pathways of PHE and substrate during ring-cleaving processes [63]. In addition, this phenolic acid has been found to occur naturally in most edible plants [64]. Furthermore, the study by Torres-Farradá et al. [11] revealed that this metabolite was the simplest and least toxic derivative, easily transformed to CO_2_ and H_2_O through biochemical reactions of microorganisms.

### 3.3. Influence of LL on N. pironii Growth and PAH Elimination

**Figure 3:**
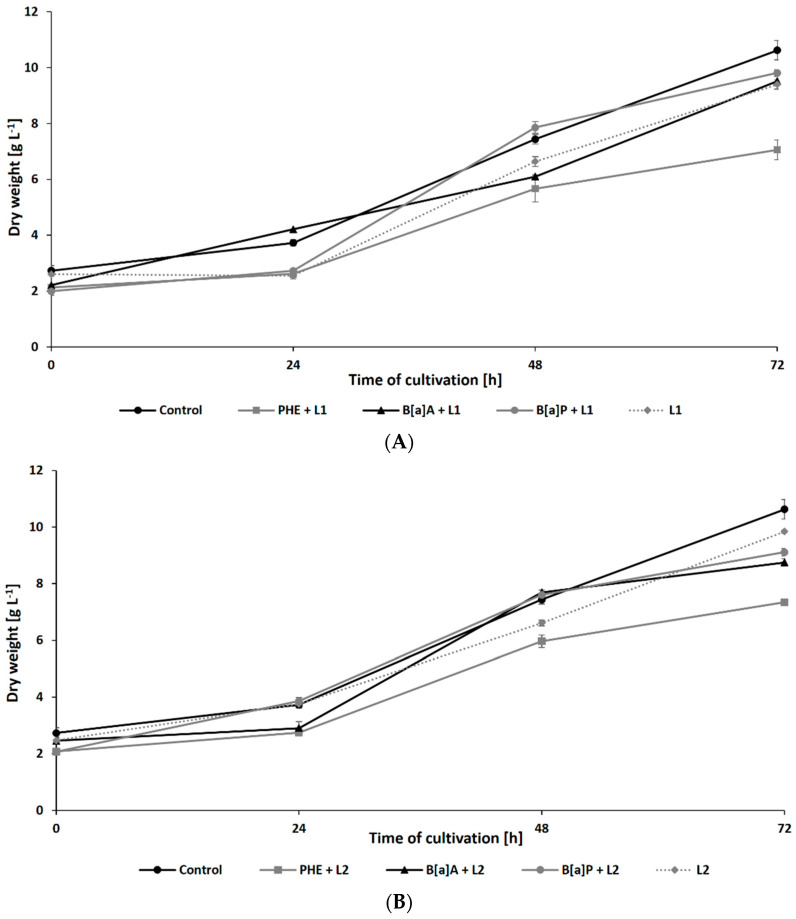
Dry weight of *N. pironii* during culture in Sabouraud medium containing PHE, B[a]A, or B[a]P (20 mg L^−1^) with 20% of L1 (**A**) or L2 (**B**).

The growth of *N. pironii* in liquid Sabouraud medium containing PHE, B[a]A, or B[a]P was also studied in the presence of leachate collected from the HW landfill. As shown in Figure 3A,B, after 72 h of incubation, the biomass content in the culture containing 20% leachate and PHE (7.06 ± 0.35 g L^−1^ for L1 and 7.34 ± 0.17 g L^−1^ for L2) was similar to that observed in the cultures containing PHE alone (7.25 ± 0.36 g L^−1^). The addition of LL did not cause a significant decrease in fungal growth after 48 h of cultivation compared to the addition of PHE alone. Both alone and in combination with LL, PHE showed a more harmful effect on *N. pironii* than B[a]A or B[a]P.

**Figure 4:**
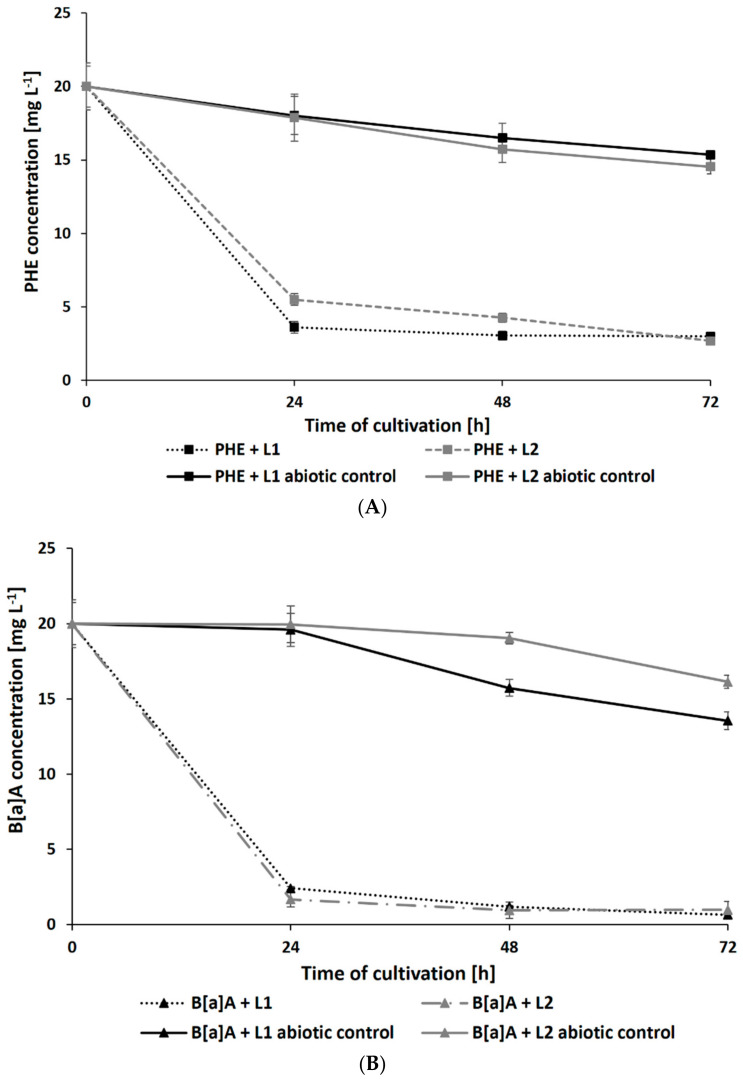

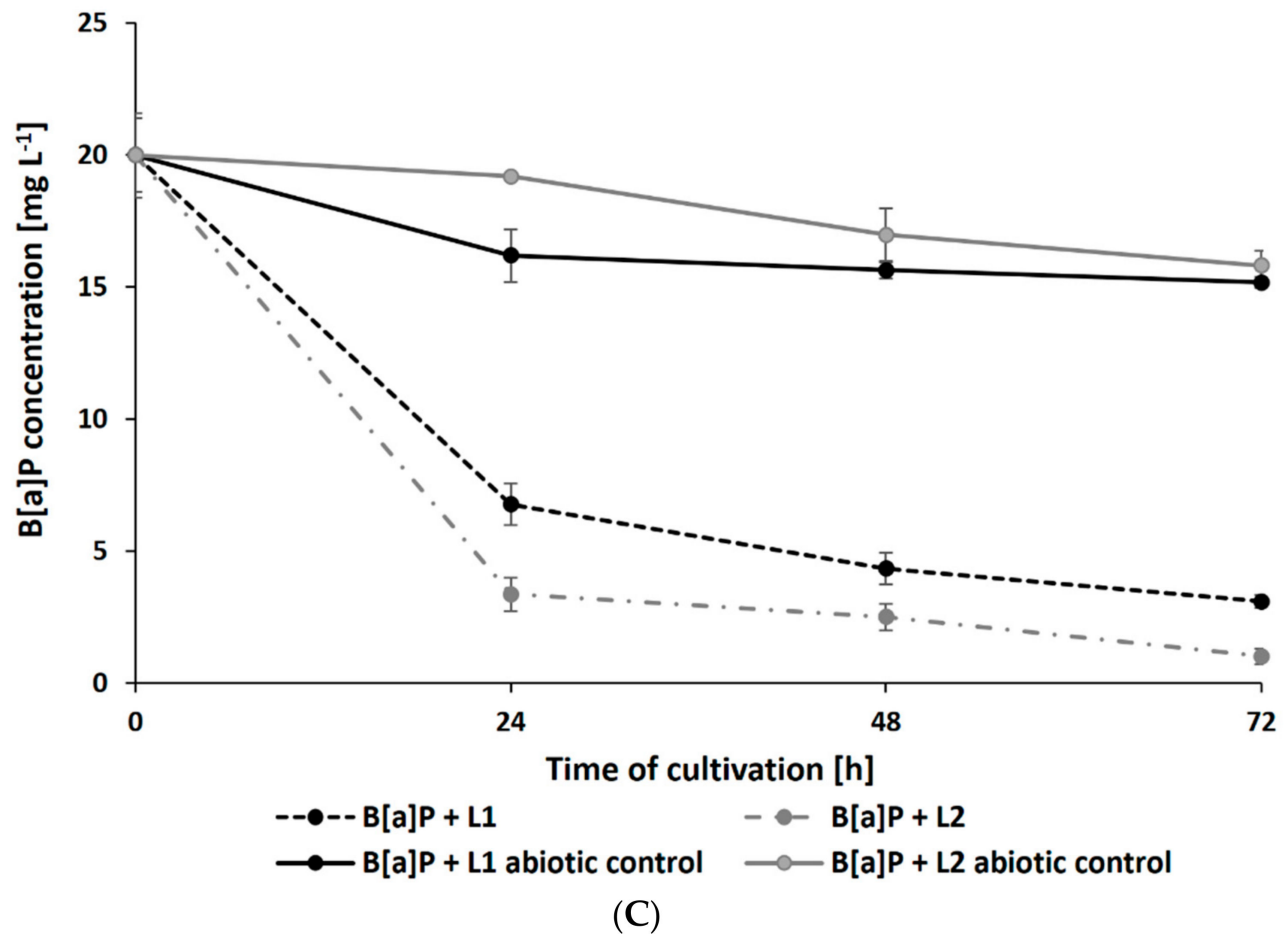
Residue of PHE (**A**), B[a]A (**B**), and B[a]P (**C**) during culture in Sabouraud medium with the addition of 20% leachate.

Surprisingly, the presence of leachates caused a significant increase in the rate of PAH degradation. After 72 h of cultivation, the residual concentration of PHE (initial concentration: 20 mg L^−1^) in samples containing L1 and L2 reached 2.69 ± 0.21 and 2.99 ± 0.19 mg L^−1^, respectively (Figure 4A). The addition of leachates also promoted the degradation of B[a]A and B[a]P by *N. pironii* (Figure 4B,C). The concentration of these compounds in cultures after 72 h of culture in the presence of L1 was 0.98 ± 0.02 and 0.65 ± 0.01 mg L^−1^ and L2 was 3.09 ± 0.25 and 1.01 ± 0.08 mg L^−1^, respectively. The higher removal efficiency of PAHs combined with LL compared to those of the individual compounds may suggest that leachates promote the transformation of these compounds. One of the possible explanations for this phenomenon is cometabolism. According to Acevedo et al. [65], cosubstrates could stimulate the secretion of catabolic enzymes and thus support the degradation of high-molecular-weight PAHs (HMW-PAHs). Janicki et al. [63] analyzed the toxicity of LL using the Phytotoxkit biotest based on the germination and growth of monocotyledonous *Sorghum saccharatum* and dicotyledonous *Lepidium sativum* and *Sinapsis alba*. In the present study, in addition to toxicity, the content of TOC was also determined. As shown in Table 1, the TOC content in L1 and L2 exceeded the permissible values. By comparing the biological oxygen demand (BOD_5_) and the COD values determined in leachate and calculating their relationship, Lisowska et al. [66] stated that it is possible to assess the susceptibility of wastewater to biodegradation. A BOD_5_/COD ratio less than 0.2 means that the wastewater is practically nonbiodegradable, while values in the range of 0.5–0.7 indicate that the wastewater can be easily biodegraded. In the study by Janicki et al. [62], the BOD_5_/COD ratio was 0.86, indicating that the leachate was readily biodegradable. LL also contains inorganic contaminants and petroleum hydrocarbons, which the fungus is known to be as a source of carbon and energy. This may stimulate catabolic processes as well as promote biodegradation and detoxication through cometabolism.

In this study, intermediate analysis after 48 and 72 h of *N. pironii* in the medium supplemented with PHE and LL revealed the presence of protocatechuic acid-TMS and phthalic acid-TMS derivatives. A protocatechuic acid-TMS derivative was also detected in cultures containing B[a]A + L1 and B[a]P + L2, indicating that *N. pironii* can convert B[a]P to less-toxic derivatives. This is an important finding because B[a]P, which consists of five fused aromatic rings, is highly stable and resistant to biodegradation. Furthermore, due to the high energy needed to break carbon–carbon bonds, B[a]P is characterized by low bioavailability [67].

### 3.4. Influence of PAHs on Membrane Condition—Lipidomic Study

The cell wall and cell membrane of microorganisms are the structures that come into direct contact with hazardous substances when they surround the cell. Microbial cell membranes play a dual role in microorganism–pollutant interactions. First, they transport pollutants inside the cell, after which the contaminants pass through the cell membranes via ion channels, self-diffusion, or transmembrane carriers and undergo intracellular changes, including complete degradation and detoxification. Second, membranes, together with the cell wall, act as a barrier that protects cells from the toxic effects of contaminants. By changing their structure, microbial membranes facilitate or limit the penetration of various substances into and out of the cell [68,69]. Membrane lipids are particularly sensitive to toxic chemicals in the surrounding environment. It has been well documented that toxic compounds induce qualitative and quantitative changes in the composition of membrane-forming lipids and thus disrupt the integrity of the cell membrane [70,71,72]. Omic methods, including lipidomics, have been used over the years to explore the mechanisms through which fungi adapt to stressful conditions.

In this study, an LC–MS/MS analysis was performed to investigate the changes that occur in the composition of membrane lipids due to the presence of PAHs in the growth medium. The analysis focused on determining the qualitative and quantitative composition of phospholipids, which are critical structural components of fungal cell membranes. Phospholipids can be divided into various classes, namely phosphatidylcholine (PC), phosphatidylethanolamine (PE), phosphatidylserine (PS), phosphatidylinsitol (PI), phosphatidylglycerol (PG), and phosphatidic acid (PA). The results of the analysis revealed that PC and PE are the primary components of the *N. pironii* membrane (Table S1, Supplementary Materials). However, their contribution to the total phospholipid pool varied depending on the culture conditions. At 24 h of cultivation, the PC content in the control cultures (containing neither PAH nor LL) was 36.15 ± 2.18% (Table 3), while it was slightly higher in the presence of leachate (20%), and was 59.98 ± 1.75% and 68.43 ± 3.45%, respectively, in the cultures grown in the presence of PHE and PHE + leachate. Changes in PC and PE influenced the PC/PE ratio, which reflects the degree of integrity and permeability of the cell membrane and can be influenced by environmental conditions [69,73]. An increase in PC content in the lipid bilayer stabilizes it, while an increase in PE, a nonlayered lipid that forms a hexagonal phase, reduces the fluidity of the membrane and increases its permeability, leading to loss of cell integrity [73]. In *N. pironii* cultures incubated with PHE alone or in combination with leachate, the PC/PE ratio was, respectively, more than 2 and 4 times higher than that in the control cultures or in the cultures containing only leachate. Interestingly, such differences were observed only in cultures containing PHE. In cultures containing B[a]P or B[a]A instead of PHE, the PC/PE ratio only slightly differed from that of the control cultures. An increase in the PC/PE ratio was found in *C. elegans* cells cultured in the presence of lipophilic tributylin (TBT) [74]. In another study, an increase in the PC/PE ratio was observed in *Metarhizium robertsii* cultures exposed to atrazine [75] or *Beauveria bassiana* cultures exposed to insecticides λ-cyhalothrin, α-cypermethrin, and deltamethrin [76]. On the other hand, a study showed a decrease in the PC/PE ratio, possibly leading to a decrease in membrane fluidity in the biomass of *Trichoderma harzianum* IM 0961 exposed to 2,4-D [72] or *M. robertsii* treated with TBT [70]. This suggests that microorganisms use different strategies to alter phospholipid membranes to maintain cell stability in the presence of various environmental contaminants. An increase in the PC/PE ratio has been observed in more-resistant fungi that are capable of maintaining the integrity of the lipid bilayer. In turn, the decrease in film fluidity resulting from the reduction in the PC/PE ratio allows for the gradual absorption of pollutants and their intracellular degradation, which is possible in the presence of low concentrations of pollutants or compounds of moderate toxicity [77]. In the study by Shon et al. [78], the bacterial strain *Sphingopyxis soli* KIT-001 cultivated in the presence of PHE showed an accelerated degradation of this compound under aerobic conditions. The level of PC and PG classes was also observed to increase, while that of PE and PA classes decreased significantly. However, data explaining the alteration occurring in the filamentous fungal membrane in response to PAHs are limited.

**Figure 5:**
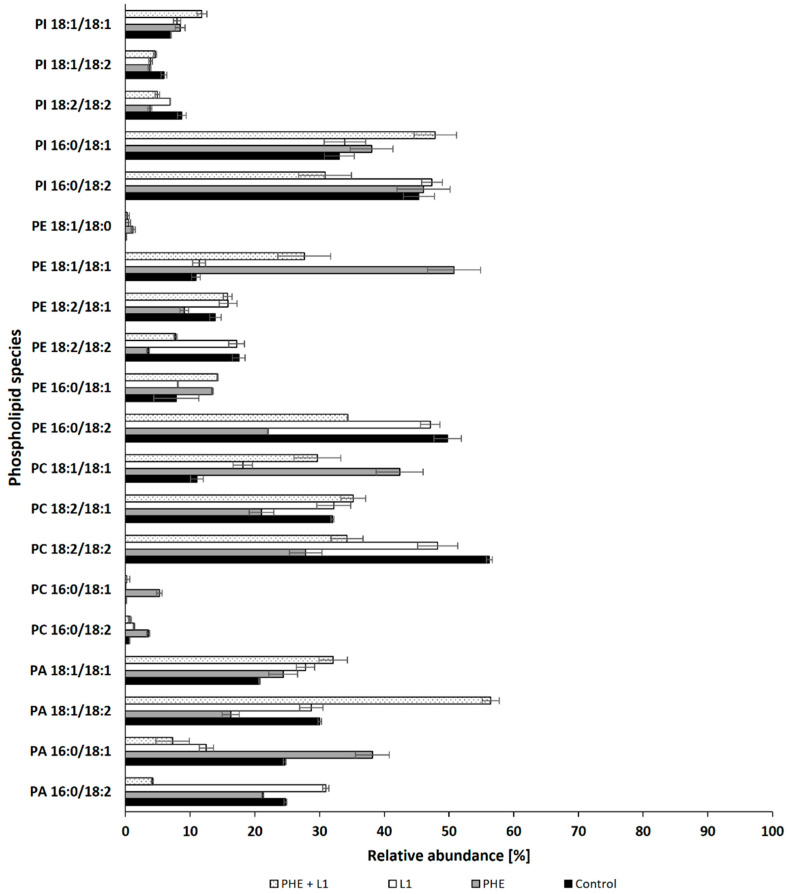
Relative abundances (%) for the main species of phospholipids in cultures of *N. pironii* supplemented with PHE, L1, or PHE + L1.

On the basis of the obtained results, it can be concluded that PHE influences the stability of the *N. pironii* membrane, which was manifested by an increase in the content of PC, enhancing the membrane integrity. These findings, together with observations regarding fungal growth (strong growth limitation in the presence of PHE), indicate that among the compounds tested, PHE caused the highest toxicity to the fungus. This was confirmed by the analysis of individual phospholipid species. The properties of biological membranes depend not only on the relative proportions of phospholipids but also on the degree of unsaturation of fatty acids in the hydrophobic part of the lipid bilayer. Analysis of the distribution of hydrocarbon chains of phospholipid fatty acids (PLFAs) showed that palmitic acid (C16:0) was the predominant saturated fatty acid, and oleic (C18:1) and linoleic acid (C18:2) were the main unsaturated species (Figure 5). In the presence of PHE, a significant decrease was observed in the content of PC 18:2/18:2; PE 18:2/18:2, and PE 18:2/18:1, accompanied by an increase in saturated forms such as PC 16:0/18:2 and PC 16:0/18:1. This confirms that PHE improves the integrity of the fungal membrane. Bernat et al. [74] observed a similar effect in the mycelium of the zygomycete *fungus C. elegans* exposed to tributyltin. Heavy metal ions have been shown to change the saturation of phospholipids in fungal hyphae. For example, cadmium and nickel ions enhanced phospholipid saturation in the mycelium of *Paecilomyces marquandii,* representing the Ascomycota cluster [44]. It was concluded that increased saturation allowed the fungus to maintain adequate membrane fluidity while mitigating the stress induced by exposure to Cd and Ni ions. Similarly, in the present study, changes in the lipid composition may have favored the fungus to adapt to unfavorable conditions. This phenomenon seems to be a universal strategy for adapting fungi belonging to different phyla.

**Figure 6:**
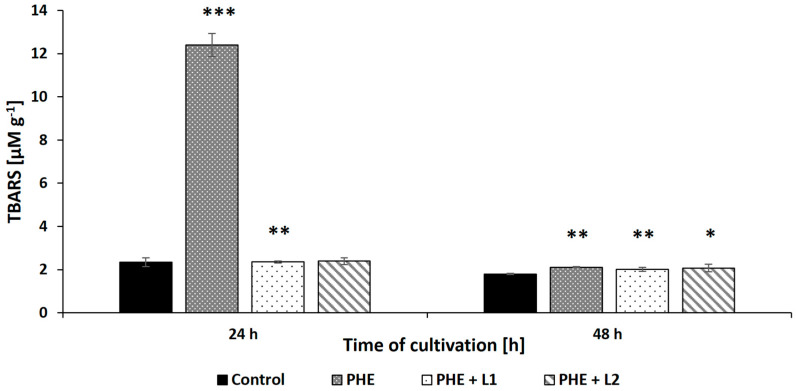
The TBARS content in the biomass of *N. pironii* exposed to PHE, L1, or PHE + L1. Data represent mean ± SE. * *p* ≤ 0.05, ** *p* ≤ 0.01, *** *p* ≤ 0.001.

Another parameter indicating the cellular changes resulting from exposure to PHE and/or leachate is the content of TBARS, which are markers of lipid peroxidation. As illustrated in Figure 6, exposure of the *N. pironii* mycelium to PHE (20 mg L^−1^) caused a statistically significant increase in the level of TBARS (*p* < 0.01) from 2.34 ± 0.21 to 12.15 ± 0.53 μM g^−1^ wet biomass during the first 24 h of cultivation. Surprisingly, the TBARS content in the biomass decreased after 48 h of incubation and was at a similar level in all tested samples (between 1.8 ± 0.03 and 2 ± 0.08 μM g^−1^). The highest TBARS content was marked in the 2,4-D-treated mycelium of *T. harzianum* than in the untreated fungus [72]. Jasińska et al. [60] also observed an increase in the level of TBARS in the mycelium exposed to BPA compared to *M. roridum* biomass cultured without xenobiotics. In the present study, the level of lipid peroxidation initially increased under the influence of PHE and then decreased to the level of the control, indicating that *N. pironii* adapted to stress and/or detoxified the pollutant after its biotransformation to less-toxic intermediates.

## 4. Conclusions

This study demonstrated that the ascomycete fungus *N. pironii*, isolated from a soil collected from the landfill of the former “Boruta” Dye Industry Plant in Zgierz (Poland), eliminated three-, four-, and five-ring PAHs from the growth medium. The fungal strain rapidly transformed the compounds tested in the presence of toxic LL collected from the area of the former “Boruta” Dye Industry Plant in Zgierz, which is also the site of fungal isolation. The observed findings confirmed the cometabolic nature of PAH elimination. LL is a mixture of organic and inorganic compounds and is characterized by high BOD_5_/COD. Thus, the conversion of PHE, B[a]A, and B[a]P was substantially accelerated during the growth of the fungus in the liquid medium. Furthermore, the identified intermediates were less toxic than the parent compounds. Additionally, PHE influenced the stability of the fungal membrane, which was manifested by changes in the classes of phospholipids, including an increase in PC content that improves membrane integrity and a simultaneous decrease in PLFA unsaturation. The results also showed enhanced lipid peroxidation, indicated by an enhanced level of TBARS in the PHE-treated mycelium. Lipidomic analysis revealed that *N. pironii* modulates its membrane composition to stabilize the cell and adapt to PAH-induced stress conditions. The present study suggests that knowledge about the course of biodegradation, the nature of the intermediates formed, and the mechanism of fungal adaptation to unfavorable conditions can be beneficial in further research with the aim of developing methods for the elimination of pollutants of multiple components. Since social and economic development leads to an intensification in generating waste, it is extremely important to decrease the amount using properly selected methods. The suitable selection of management and storage methods is also crucial to avoid releasing hazardous waste in landfills to the environment and thus posing the risk to society. the use of ascomycete fungus *N. pironii* belonging to Ascomycota in PAH elimination was also noteworthy in this work. The obtained results may contribute to broadening the knowledge regarding PAHs removal by fungi, especially Ascomycota. To sum up, the results presented in this work in combination with other conventional physical/chemical methods might find an application in PAH removal on a large scale.

## Figures and Tables

**Table 1:** Summary of the most important results for 2017–2021 from the study of leachate from the Zgierz landfill based on the reports supported by the Voivodeship Inspectorate of Environmental Protection in Łódź (Poland) of 07 XI 2021.

Date of Sampling	COD_Mang_ (N = 125)		TOC (N = 30)		Iron (N = 10)		Petroleum Hydrocarbons (N = 15)	
	mg L^−1^ O_2_		mg L^−1^ C		mg L^−1^ Fe		mg L^−1^	
	L1	L2	L1	L2	L1	L2	L1	L2
17 X 2017	564 ± 150	568 ± 151	787 ± 118	753 ± 113	61.5 ± 11.7	58.4 ± 11.1	3.01 ± 0.81	3.80 ± 1.03
09 X 2018	247 ± 66	251 ± 67	540 ± 99	525 ± 101	27.3 ± 5.2	26.2 ± 5.0	5.40 ± 1.36	14.0 ± 3.5
09 IV 2019	460 ± 122	488 ± 130	725 ± 133	765 ± 140	22.3 ± 4.2	24.3 ± 4.6	1.02 ± 0.26	0.60 ± 0.15
11 VII 2019	520 ± 138	494 ± 131	660 ± 176	672 ± 179	13.2 ± 2.5	13.1 ± 2.5	6.13 ± 1.54	4.82 ± 1.21
21 V 2020	426 ± 113	428 ± 114	799 ± 146	798 ± 146	23.8 ± 4.5	23.1 ± 4.4	3.12 ± 0.79	2.08 ± 0.52
16 XI 2020	522.8 ± 138.5	348.8 ± 92.4	826 ± 151	1040.5 ± 190.4	28.86 ± 5.48	30.88 ± 5.87	23.7 ± 7.6	7.2 ± 2.3
09 III 2021	448 ± 119	460.8 ± 122.6	274.2 ± 50.2	294.7 ± 53.9	57 ± 11	31 ± 5.9	52.2 ± 16.7	1.91 ± 0.61
07 IX 2021	444 ± 118	392 ± 104	437 ± 80	372 ± 68	39.1 ± 7.4	32.9 ± 6.3	17.0 ± 5.4	14.8 ± 4.7

N—limit values, according to the Regulation of the Minister of Maritime Economy and Inland Navigation of 12 July 2019 on substances particularly harmful to the aquatic environment and the conditions to be met when discharged sewage into waters or ground, as well as when discharging rainwater or meltwater into waters or into aquatic devices. Dz. U. 2019 poz. 1311. https://isap.sejm.gov.pl/isap.nsf/download.xsp/WDU20190001311/O/D20191311.pdf (accessed on 21 May 2022).

**Table 2:** Metabolites of degradation of PHE, B[a]A, and B[a]P obtained after *N. pironii* cultivation in the presence of leachate.

Compound Name	Chemical Structure	Fragment Ion *(m/z)*	Precursor Ion (*m/z*)
protocatechuic acid (tms)	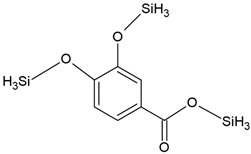	193 281 355	370
phthalic acid (tms)	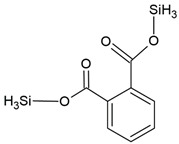	147 221 295	310
terephthalic acid (tms)	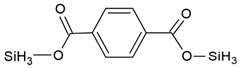	221 251 295	310
p-hydroxyphenylacetic acid (2 tms)	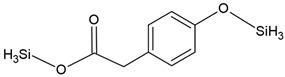	252 281	296

**Table 3:** The composition of phospholipids (PL) and PC/PE ratio determined in *N. pironii* cells after 24 h of cultivation with PHE, L1, or PHE + L1, or without the compounds tested. PA, phosphatidic acid; PE, phosphatidylethanolamine; PC, phosphatidylcholine; PG, phosphatidylglycerol; PI, phosphatidylinositol; PS, phosphatidylserine. Data represent mean ± SE. * *p* ≤ 0.05, ** *p* ≤ 0.01.

PL Class	Control Biotic	L1	PHE	PHE + L1	B[a]A	B[a]A + L1	B[a]P	B[a]P + L1
PA	0.65 ± 0.02	1.96 ± 0.11 **	0.42 ± 0.02 *	0.19 ± 0.01 **	0.71 ± 0.03	1.20 ± 0.04 **	0.43 ± 0.02	0.88 ± 0.07
PC	36.15 ± 2.18	40.60 ± 2.54 *	59.98 ± 1.75 **	68.43 ± 3.45 **	45.11 ± 1.45 **	42.06 ± 3.36 **	40.51 ± 3.54 *	47.67 ± 2.39 *
PE	58.08 ± 2.36	52.45 ± 3.17 *	38.48 ± 2.14 **	24.51 ± 1.19 **	51.26 ± 3.36 *	48.09 ± 1.56 **	55.70 ± 4.25	45.49 ± 3.35 *
PI	5.12 ± 0.41	4.99 ± 0.18	1.12 ± 0.04 **	6.88 ± 0.23	2.92 ± 0.14	8.65 ± 0.37 **	3.37 ± 0.21	5.96 ± 0.46
PC/PE	0.62 ± 0.03	0.77 ± 0.03	1.56 ± 0.07 *	2.79 ± 0.25 **	0.88 ± 0.05	0.87 ± 0.04	0.73 ± 0.04	1.05 ± 0.09

## Data Availability

Data are contained within the article.

## Supplementary Materials

The following supporting information can be downloaded at: https://www.mdpi.com/article/10.3390/ijerph192113997/s1, Figure S1: Mass spectra of qualitative analysis of PHE, B[a]A and B[a]P degradation by *N. pironii* cultivated with the presence of leachate; Table S1: The phospholipids composition and PC/PE ratio determined in the *N. pironii* cells after 24, 48, and 72 h of cultivation with PHE, L1 or PHE + L1, or without the tested compounds.
