# Supplementary material for: Accelerated PAH Transformation in the Presence of Dye Industry Landfill Leachate Combined with Fungal Membrane Lipid Changes
Source: Int J Environ Res Public Health. 2022 Oct 27;19(21):13997. doi: 10.3390/ijerph192113997 (PMC9654376; doi:10.3390/ijerph192113997)
Supplement: Supplementary file 1 [file ijerph-19-13997-s001.zip › Table S1.pdf]

Table S1. The phospholipids composition and PC/PE ratio determined in the *N. pironii* cells after 24, 48, and 72 h of cultivation with PHE, L1 or PHE + L1, or without the tested compounds.

	Phospholipid class	Control biotic	L1	PHE	PHE + L1	B[a]A	B[a]A + L1	B[a]P	B[a]P + L1
**24 h**	**PA**	0.65±0.02	1.96±0.11	0.42±0.02	0.19±0.01	0.71±0.03	1.20±0.04	0.43±0.02	0.88±00.7
	**PC**	36.15±2.18	40.60±2.54	59.98±1.75	68.43±3.45	45.11±1.45	42.06±3.36	40.51±3.54	47.67±2.39
	**PE**	58.08±2.36	52.45±3.17	38.48±2.14	24.51±41.19	51.26±3.36	48.09±1.56	55.70±4.25	45.49±3.35
	**PI**	5.12±0.41	4.99±0.18	1.12±0.04	6.88±0.23	2.92±0.14	8.65±0.37	3.37±0.21	5.96±0.46
	**PC/PE**	0.62±00.3	0.77±0.03	1.56±0.07	2.79±0.25	0.88±0.05	0.87±0.04	0.73±0.04	1.05±0.09
**48 h**	**PA**	0.43±0.02	11.60±0.91	0.53±0.04	0.11±0.01	0.46±0.03	1.05±0.58	0.34±0.02	1.21±0.11
	**PC**	40.27±3.24	36.39±2.27	68.28±5.54	70.24±6.37	48.66±3.54	48.49±3.69	47.72±2.24	57.71±5.31
	**PE**	55.25±4.98	46.56±3.39	29.06±2.68	24.68±2.21	47.97±4.11	41.18±2.98	49.42±3.87	35.57±3.36
	**PI**	4.04±0.36	5.48±0.44	2.13±0.19	4.97±0.37	2.91±0.26	9.27±0.87	2.52±0.19	5.51±0.45
	**PC/PE**	0.73±0.06	0.78±	2.35±0.19	2.85±0.21	1.01±0.04	1.18±0.14	0.97±0.06	1.62±0.13
**72 h**	**PA**	0.34±0.02	0.80±0.63	0.82±0.07	0.18±0.01	0.43±0.02	1.23±0.11	0.71±.05	1.05±0.09
	**PC**	35.04±2.7	39.90±2.11	58.35±4.9	73.64±6.54	41.44±3.37	34.90±2.94	32.49±2.74	37.10±2.,97
	**PE**	59.74±5.69	54.65±2.87	38.51±2.25	21.19±1.98	55.10±4.65	55.64±4.98	63.34±5.79	54.15±4.36
	**PI**	4.88±3.61	4.65±0.06	2.32±0.28	4.99±3.56	3.03±0.28	8.23±0.44	3.46± 0.28	7.70±0.69
	**PC/PE**	0.59±0.48	0.73±0.01	1.52±0.14	3.48±0.27	0.75±0.04	0.63±0.03	0.51±0.03	0.69±0.05
