# Supplementary figures and images for: Accelerated PAH Transformation in the Presence of Dye Industry Landfill Leachate Combined with Fungal Membrane Lipid Changes
Source: Int J Environ Res Public Health. 2022 Oct 27;19(21):13997. doi: 10.3390/ijerph192113997 (PMC9654376; doi:10.3390/ijerph192113997)

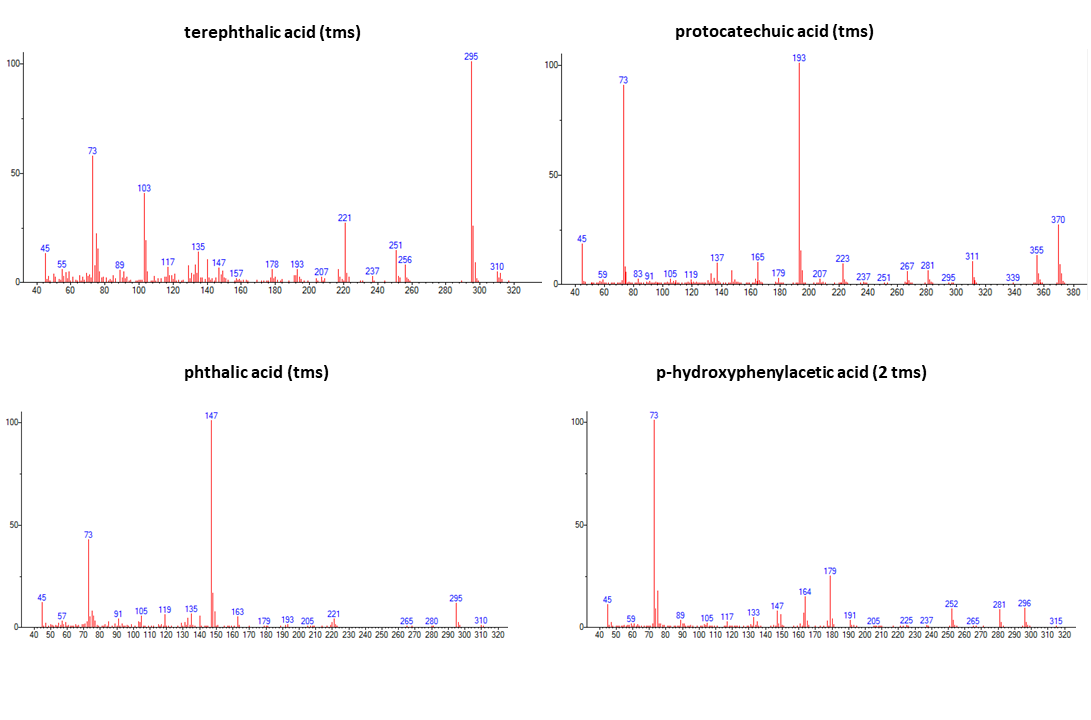

Supplement: Supplementary file 1 [file ijerph-19-13997-s001.zip › Figure S1.tif]
